# Supplementary material for: Moderate Reduction in Nitrogen Fertilizer Results in Improved Rice Quality by Affecting Starch Properties without Causing Yield Loss
Source: Foods. 2023 Jul 5;12(13):2601. doi: 10.3390/foods12132601 (PMC10341124; doi:10.3390/foods12132601)
Supplement: Supplementary file 1 [file foods-12-02601-s001.zip › foods-2460865-supplementary.pdf]

Supplemental figures and tables

Table S1 The TPA parameters of cooked rice in different nitrogen application rates

Year	Cultivars	Treatment	Adhesiveness (mJ)	cohesiveness	springiness (mm)	gumminess (g)	chewiness (mJ)
2020	Bengal	N0	0.15±0.01a	0.38±0.02a	0.65±0.04a	69.53±7.73b	0.46±0.04b
		N1	0.12±0b	0.39±0.02a	0.65±0.05a	63.08±3.27b	0.38±0.03b
		N2	0.14±0.01a	0.42±0.02a	0.70±0.04a	92.67±17.93a	0.63±0.10a
	SD505	N0	0.14±0.03a	0.42±0.02a	0.66±0.01a	79.20±5.03a	0.53±0.04a
		N1	0.12±0.01a	0.42±0.02a	0.71±0.03a	85.81±6.80a	0.58±0.06a
		N2	0.09±0b	0.40±0.02a	0.64±0.01a	85.21±14.34a	0.54±0.11a
2021	Bengal	N0	0.16±0.02a	0.41±0.02a	0.62±0.02a	86.33±4.03a	0.53±0.04a
		N1	0.11±0.02b	0.41±0.01a	0.64±0.02a	90.82±1.96a	0.58±0.02a
		N2	0.10±0.01b	0.41±0.02a	0.63±0.02a	92.29±4.52a	0.58±0.05a
	SD505	N0	0.16±0.02a	0.47±0.02a	0.75±0.05a	104.5±5.63a	0.76±0.04a
		N1	0.14±0.03ab	0.44±0.03a	0.75±0.08a	103.86±11.88a	0.72±0.10a
		N2	0.12±0b	0.46±0a	0.69±0.01a	112.31±4.56a	0.78±0.05a

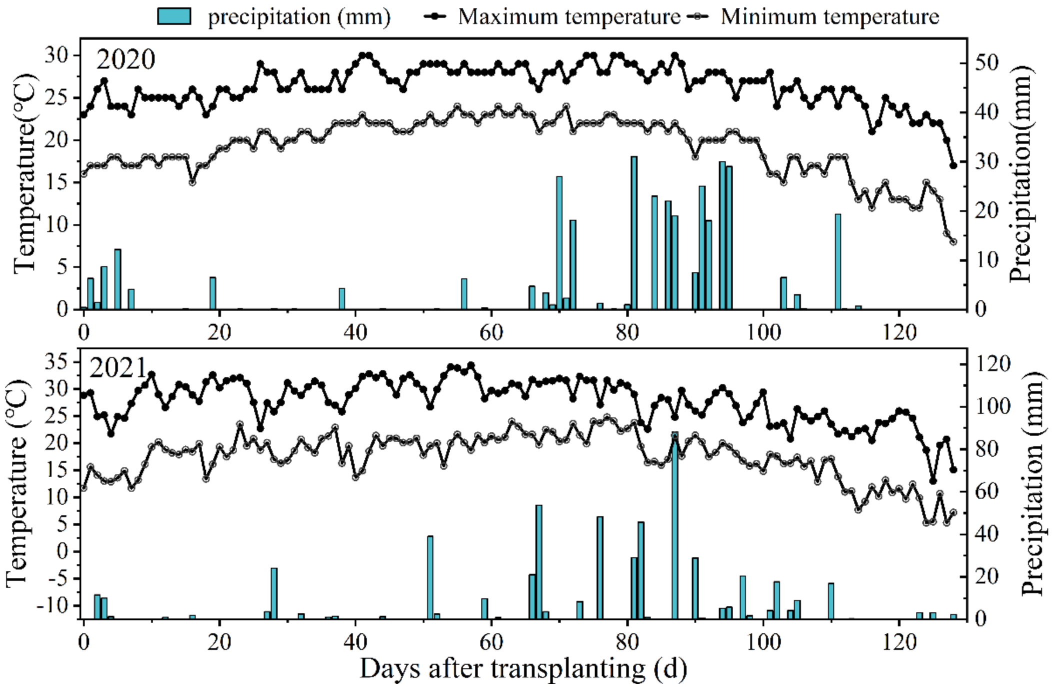

Figure S1 Temperature and precipitation in 2020 and 2021 during rice cultivation.

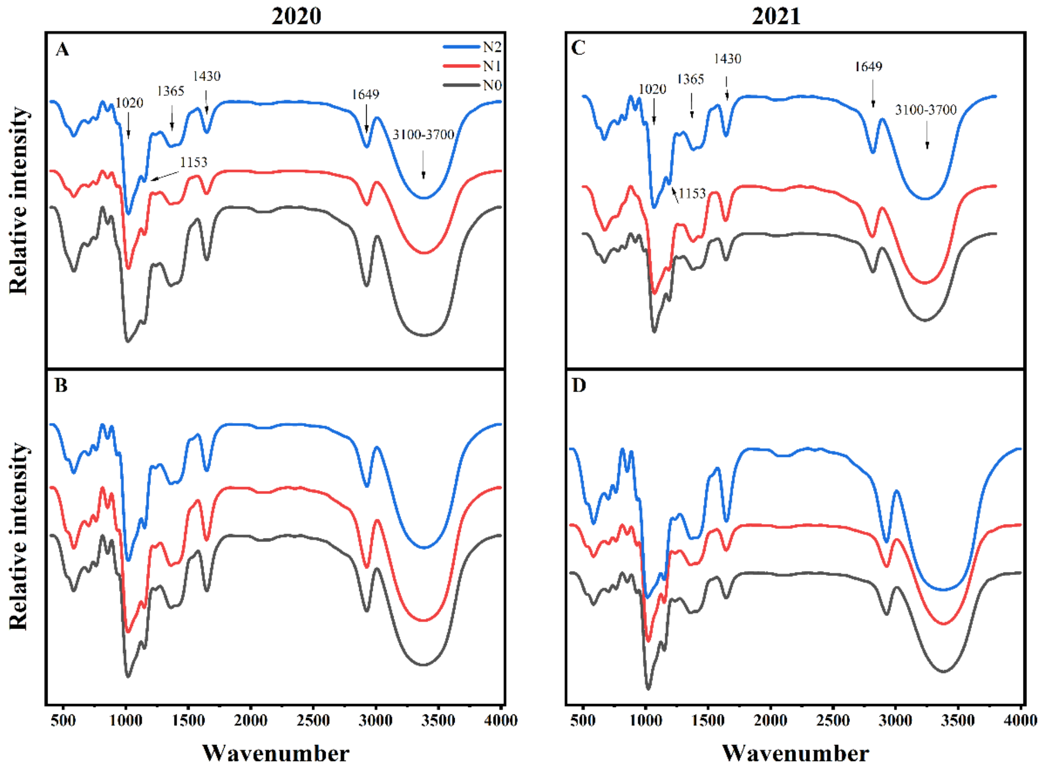

Figure S2 FTIR spectrum in different nitrogen rates from Bengal (A and C) and SD505 (B and D) in 2020 (A and B) and 2021 (C and D).

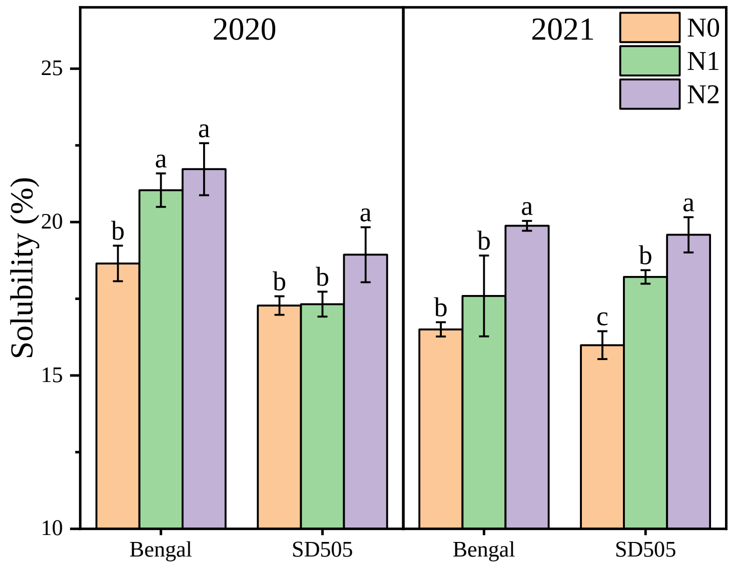

Figure S3 Solubility in different nitrogen rates from Bengal and SD505.
